# Supplementary figures and images for: Twenty-first-century demographic and social inequalities of heat-related deaths in Brazilian urban areas
Source: PLoS One. 2024 Jan 24;19(1):e0295766. doi: 10.1371/journal.pone.0295766 (PMC10807764; doi:10.1371/journal.pone.0295766)

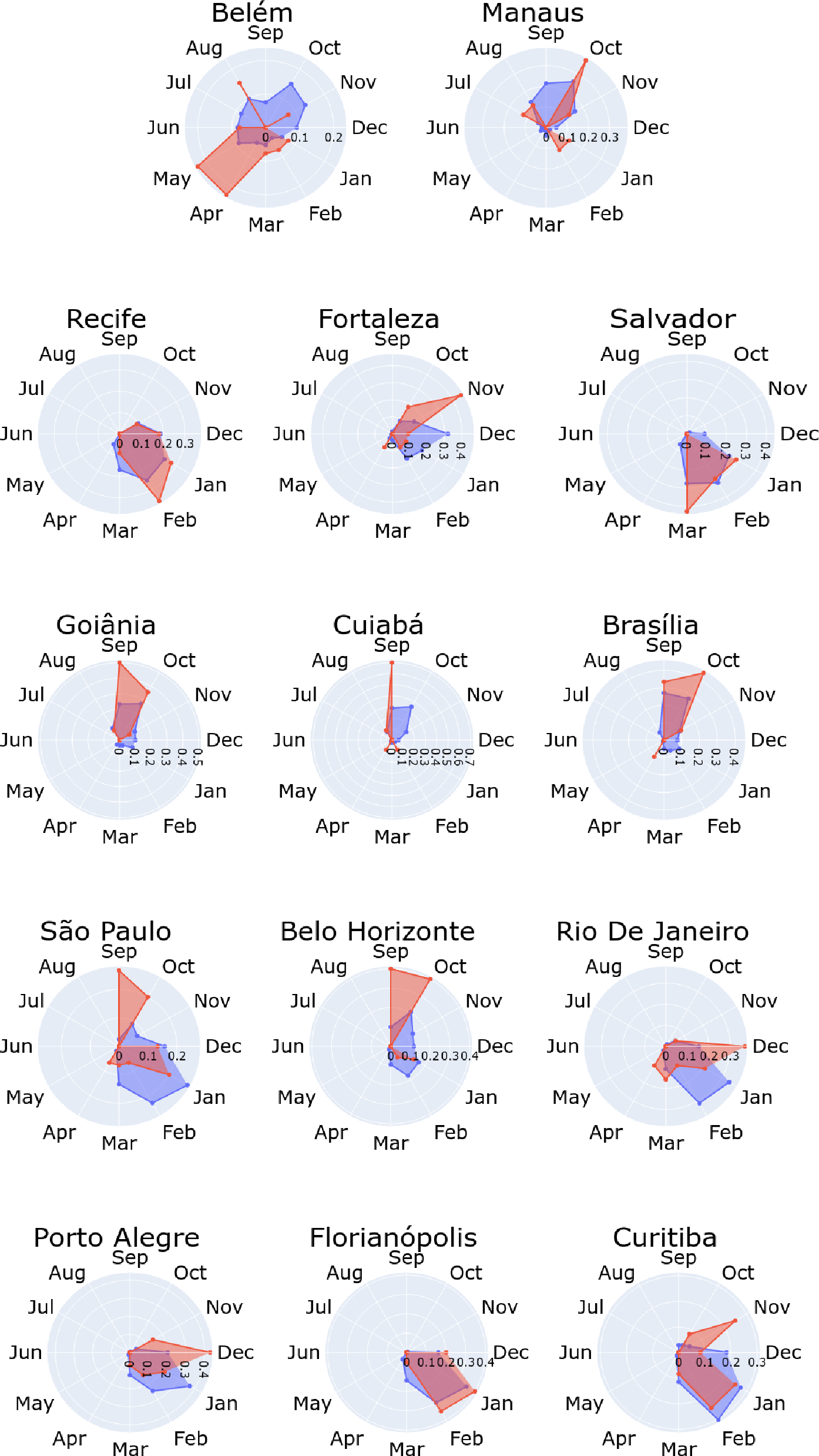

Supplement: S1 Fig — The Northern MRs are presented in the first row, Northeastern MRs are in the second row, Central-western MRs are in the third row, Southeastern in the fourth row, and Southern MRs are presented in the fifth row. (TIF) [file pone.0295766.s001.tif]

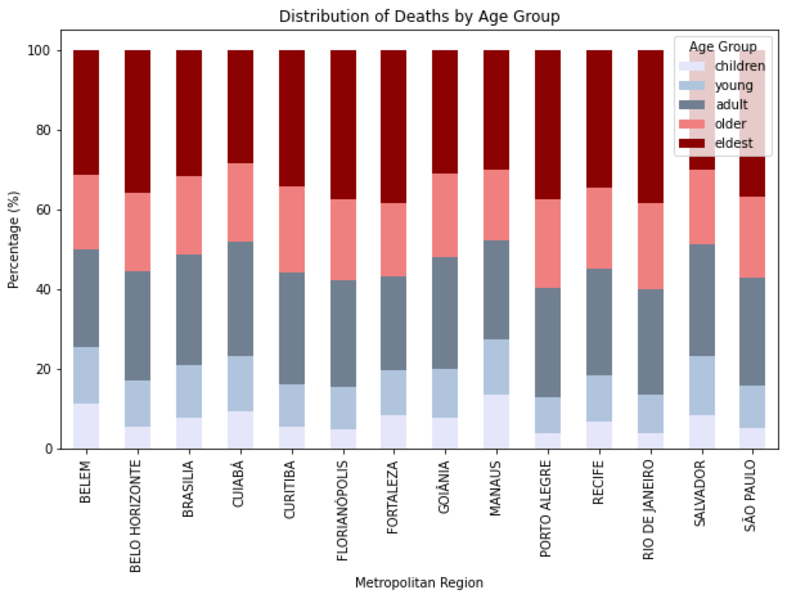

Supplement: S2 Fig — The percentage of death among older and eldest is 47.65% in the MR of Manaus, 47.99% in Cuiabá, 48.58% in Salvador, 49.92% in Belém, 51.16% in Brasilia, 51.93% in Goiânia, 54.87% in Recife, 55.57% in Belo Horizonte, 55.82% in Curitiba, 56.90% in Fortaleza, 57.06% in São Paulo, 57.65% in Florianópolis, 59.65% in Porto Alegre, and 59.90% in Rio de Janeiro. (TIF) [file pone.0295766.s002.tif]
