# Supplementary material for: Twenty-first-century demographic and social inequalities of heat-related deaths in Brazilian urban areas
Source: PLoS One. 2024 Jan 24;19(1):e0295766. doi: 10.1371/journal.pone.0295766 (PMC10807764; doi:10.1371/journal.pone.0295766)
Supplement: S1 Table — ICD-10 chapters: II (Neoplasms), IV (Endocrine, Nutritional and Metabolic Diseases), IX (Diseases of the Circulatory System), V (Mental and Behavioral Disorders), VI (Diseases of the Nervous System), X (Diseases of the Respiratory System), XI (Diseases of the Digestive System), XII (Diseases of the Skin and Subcutaneous Tissue), XIV (Diseases of the Genitourinary System). (PDF) [file pone.0295766.s003.pdf]

	ICD-10 chapters								
	**II**	**IV**	**IX**	**V**	**VI**	**X**	**XI**	**XII**	**XIV**
**Metropolitan Region**									
**BELÉM**	1.35 (1.30-1.40)<br>1777 ED	1.55(1.46-1.64)<br>954 ED	1.24(1.20-1.27)<br>1721 ED	1.77(1.32-2.39)<br>52 ED	1.88(1.66-2.12)<br>355 ED	1.34(1.28-1.39)<br>1258 ED	1.30(1.22-1.39)<br>507 ED	2.16(1.72-2.71)<br>125 ED	1.58(1.43-1.74)<br>379 ED
**BELO HORIZONTE**	1.22(1.17-1.26)<br>1002 ED	1.24(1.14-1.34)<br>267 ED	1.02(0.99-1.06)<br>145 ED	1.62(1.42-1.85)<br>217 ED	1.69(1.54-1.87)<br>441 ED	1.19(1.13-1.26)<br>461 ED	1.10(1.02-1.19)<br>134 ED	1.53(1.18-1.99)<br>49 ED	1.58(1.41-1.76)<br>300 ED
**BRASÍLIA**	1.30(1.22-1.38)<br>522 ED	1.26(1.12-1.41)<br>137 ED	1.08(1.03-1.14)<br>242 ED	1.59(1.29-1.97)<br>81 ED	2.01(1.71-2.37)<br>215 ED	1.43(1.31-1.56)<br>347 ED	1.21(1.08-1.36)<br>111 ED	13.7(3.55-52.8)<br>29 ED	1.7(1.4-2.06)<br>115 ED
**CUIABÁ**	1.28(1.10-1.50)<br>79 ED	1.85(1.43-2.4)<br>75 ED	1.35(1.19-1.53)<br>151 ED	***	2.50(1.59-3.93)<br>40 ED	1.69(1.38-2.09)<br>98 ED	1.33(0.99-1.80)<br>25 ED	***	***
**CURITIBA**	1.21(1.13-1.29)<br>355 ED	1.29(1.13-1.46)<br>122 ED	1.11(1.05-1.18)<br>247 ED	1.64(1.21-2.22)<br>43 ED	1.79(1.50-2.13)<br>155 ED	1.24(1.12-1.36)<br>168 ED	1.16(1.03-1.32)<br>77 ED	2.16(0.95-4.92)<br>10 ED	1.41(1.14-1.73)<br>62 ED
**FORTALEZA**	1.31(1.24-1.39)<br>631 ED	***	1.17(1.12-1.23)<br>504 ED	***	1.81(1.55-2.10)<br>214 ED	1.46(1.36-1.58)<br>515 ED	1.30(1.17-1.45)<br>178 ED	2.39(1.61-3.55)<br>49 ED	1.58(1.31-1.9)<br>105 ED
**GOIÂNIA**	1.25(1.19-1.33)<br>562 ED	1.22(1.09-1.36)<br>128 ED	1.08(1.03-1.13)<br>304 ED	***	1.73(1.48-2.02)<br>180 ED	1.31(1.23-1.40)<br>478 ED	1.07(0.98-1.17)<br>66 ED	2.83(1.52-5.27)<br>25 ED	1.52(1.31-1.78)<br>140 ED
**MANAUS**	1.37(1.29-1.45)<br>711 ED	1.75(1.58-1.94)<br>444 ED	1.25(1.18-1.32)<br>525 ED	***	1.80(1.47-2.21)<br>116 ED	1.25(1.15-1.36)<br>246 ED	1.18(1.05-1.33)<br>98 ED	***	1.29(1.09-1.53)<br>67 ED
**PORTO ALEGRE**	1.12(1.07-1.16)<br>565 ED	1.41(1.30-1.53)<br>413 ED	1.19(1.14-1.23)<br>1037 ED	1.50(1.22-1.85)<br>73 ED	1.82(1.64-2.02)<br>463 ED	1.30(1.23-1.38)<br>618 ED	1.06(0.97-1.15)<br>60 ED	1.77(1.12-2.78)<br>23 ED	1.55(1.35-1.76)<br>197 ED
**RECIFE**	1.31(1.23-1.40)<br>553 ED	1.15(1.04-1.27)<br>109 ED	1.18(1.13-1.24)<br>622 ED	1.55(1.10-2.19)<br>29 ED	2.09(1.74-2.49)<br>193 ED	1.39(1.29-1.49)<br>512 ED	1.17(1.06-1.29)<br>131 ED	4.02(2.55-6.33)<br>70 ED	1.87(1.61-2.16)<br>241 ED
**RIO DE JANEIRO**	1.08(1.05-1.10)<br>912 ED	1.16(1.11-1.21)<br>748 ED	1.16(1.14-1.18)<br>3324 ED	1.21(1.07-1.36)<br>107 ED	1.48(1.38-1.58)<br>677 ED	1.27(1.23-1.31)<br>2139 ED	1.05(1.00-1.10)<br>161 ED	1.62(1.39-1.89)<br>158 ED	1.5(1.42-1.59)<br>1034 ED
**SÃO PAULO**	1.11(1.09-1.13)<br>2826 ED	1.14(1.10-1.18)<br>768 ED	1.08(1.07-1.10)<br>3424 ED	1.32(1.23-1.43)<br>389 ED	1.46(1.39-1.52)<br>1438 ED	1.23(1.21-1.26)<br>3440 ED	1.06(1.03-1.09)<br>465 ED	1.56(1.37-1.77)<br>223 ED	1.46(1.39-1.53)<br>1357 ED
