# Supplementary material for: Twenty-first-century demographic and social inequalities of heat-related deaths in Brazilian urban areas
Source: PLoS One. 2024 Jan 24;19(1):e0295766. doi: 10.1371/journal.pone.0295766 (PMC10807764; doi:10.1371/journal.pone.0295766)
Supplement: Attachment — Submitted filename: PONE-D-23-10924 - Answer to reviewer comments #2.docx [file pone.0295766.s005.docx]

**PONE-D-23-10924 - Answer to the second round of reviewer’s comments**

*Twenty-first-century demographic and social inequalities of heat-related deaths in Brazilian urban areas*

To organize all individual comments and answers plus actions, we highlighted the comments and answers in the following way: Blue text shows the reviewer comments [R1], [R2, and [R3], and black text shows the authors' response [A1], [A2], and [A3].

**Reviewer #1:**

**[R1 General Comment]:** The overall manuscript is almost ready for publication. Minor suggestions/reviews should be addressed as it follows:

[A1-General Comment] We thank the reviewer for the positive evaluation of the overall manuscript. We addressed the minor suggestions and reviews accordingly, as follows:

**[R1.1]** Concerning races. Currently, it is no longer reliable to classify diseases by 'race' in hospital discharges, given the great homogeneity of our species (Tishkoff & Kidd,2004, and Lorusso & Bachini, 2015). D. Roberts (2011) and J Dupré (2008) emphasize that the biological term for race is a form of racism, and it is a mistake to accept that races, in the plural, exist. The Genome Project even states the following: The completion of the Human Genome Project in 2003 confirmed humans are 99.9% identical at the DNA level and there is no genetic basis for race. Pena (2005) presented that only 0.0005 to 0.001% of our genome is characterized by the phenotype (the correct word for those 'differences', rather than 'races'). Therefore I suggest that you keep your finds but use the above statements to explain the readers.

**[A1.1]** Considering the reviewer's comment concerning race/color/ethnicity, we would like to stress that we do not suggest in any part of the manuscript that skin color, in isolation, can affect how the body responds to extreme heat. As discussed in sections 2.2, 3.3, and 4.3, and as suggested by the manuscript's title, our study uses the variable race/color/ethnicity as a proxy for social markers that drive the population's vulnerability and exposure, influencing heat-related deaths in Brazilian urban areas. The social position of individuals affects their health and vulnerability to hazards, and this variable (race/color/ethnicity) has been widely used as a proxy in studies on health and healthcare inequalities ([Travassos and Willians,](https://www.scielo.br/j/csp/a/zd6YRvbF56P9WgRJFwRq8hg/?format=pdf&lang=en) 2004; [Silva et al., 2020](https://www.scielo.br/j/reben/a/nMTkjYhjBNwbqmQCDZNPKzM/); [Cobo et. al, 2021](https://doi.org/10.1590/1413-81232021269.05732021); [Dantas-Silva et al., 2023](https://www.ncbi.nlm.nih.gov/pmc/articles/PMC10281767/#:~:text=In%20Brazil%2C%20infection%20and%20death,those%20with%20low%20socioeconomic%20status.&text=Mortality%20from%20COVID%2D19%20was,other%20women%20in%20our%20country.); [Filha et al, 2016](https://www.sciencedirect.com/science/article/pii/S0165032715306789?casa_token=-9LiNLPpFcUAAAAA:oTTEVKJn86ISD9uvp7XUhOi4rdJr6MFct26lR_n1AFP5_wGt8UaTXRkrDlMQZVTNZ58E5TWbP-o); [Oliveira et. al, 2020](https://www.scielo.br/j/csp/a/QvQqmGfwsLTFzVqBfRbkNRs/?lang=pt); [Rebouças, et. al, 2022](https://www.thelancet.com/journals/langlo/article/PIIS2214-109X(22)00333-3/fulltext)). Such studies have highlighted race/color/ethnicity as a determining factor for inequalities in access to healthcare and other health indicators. Race/color/ethnicity reflects the relationships between individuals and society and is intrinsically connected to socioeconomic heterogeneities, such as income, living conditions, social mobility, access to university, political participation, and other social markers indirectly influencing health ([Muniz, 2010](https://www.scielo.br/j/rsocp/a/MWPjc8LtLcPrcg4RfTcPXpv/abstract/?lang=en)). Understanding the role played by race/color/ethnicity in revealing socioeconomic differences related to the risk of illness and death can contribute to the development of policies aimed at reducing health inequalities. Taking this into account, we modified some parts of the manuscript to clarify the role of race/color/ethnicity as a relevant socioeconomic indicator from the point of view of the impact of heatwaves on mortality rates, as follows:

- In Material and Methods, Section 2.2, pg. 7, we added the following sentence:

*“Intersectionality approaches that consider socioeconomic and demographic markers, such as proposed here, are crucial to understanding how different social groups experience differential vulnerability to climate hazards. According to the IPCC Sixth Assessment Report - Impacts, Adaptation, and Vulnerability (*[*Birkmann et al., 2022*](https://www.ipcc.ch/report/ar6/wg2/chapter/chapter-8/)*), age, gender, class, race, and ethnicity are reinforcing social phenomena that shape social inequalities and, therefore, intersect with climate hazards and vulnerability. From this point of view, both race and educational level might be interpreted as determinants of social inequalities and a factor that leads to heat-related risk of illness and mortality.”*

- In the Introduction, pg. 5, we slightly modified the aims as follows:

*"It is aimed to evaluate the interplay of intersectionality and vulnerability to heat considering demographics and socioeconomic heterogeneities, using gender, age, race, and educational level as proxies."*

- In section 4.3, pg. 19, we clarify, pointing to race and education as socioeconomic factors, as follows:

*"Disparities in the risk of heat-related death associated with socioeconomic factors (race and education level) were also observed across the MRs. "*

- In the Abstract, we modified the following sentence:

*"We estimated excess mortality during HWs through the ratio between observed and expected deaths (O/E) during the events identified, including the leading causes of heat-related excess death. Moreover, we assessed the interplay of intersectionality and vulnerability to heat, considering demographics and socioeconomic heterogeneities, using gender, age, race, and educational level."*

- In Discussion, pg. 18, we changed the subtitle "4.3 - Key groups most sensitive to heat-related risk in Brazil" to:

*"4.3 - The interplay of intersectionality and vulnerability on heat-related risk in Brazil."*

- In Discussion, pg. 18, we add the following phrase:

*"Our results shed light on the relationships between demographic and socioeconomic heterogeneities and heat exposure in Brazil."*

Moreover, the following paragraph (pg. 20) was improved:

*"The racial disparities in heat-related mortality observed here reinforce how the discussion about health effects due to extreme heat in urban areas intersects the debate on environmental racism, which refers to the disproportional burden of environmental hazards among the racial/ethnic groups (Farbent, 2022; Perez et al., 2015; Sun et al., 2022; Watkins and Gerrish, 2018; Baptista and Santos, 2022). It is essential to point out that, in this context, race/skin color should not be considered as a biological factor but as a social variable that determines, among other aspects, vulnerability and health risk as a consequence of discrimination, exclusion, and racial inequalities socially produced by historical and cultural processes (*[de Araújo et al., 2009](https://doi.org/10.1590/S1414-32832009000400012)*). Our results corroborate with previous studies on intersectionality and vulnerability to climate hazards worldwide (Bailey et al., 2017; Ellena et al., 2020; Gronlund, 2014; Marí-Dell’Olmo et al., 2019; Vandentorren et al., 2006). In the U.S., for example, black people presented higher mortality rates for cardiovascular diseases due to increasing levels of PM2.5 (Ma et al.,* [*2023*](https://www.nature.com/articles/s41562-023-01694-7)*) and higher exposure to surface urban heat islands (Hsu et al.,* [*2023*](https://www.nature.com/articles/s41467-021-22799-5)*), compared to non-Hispanic-white-people. In Brazil, several studies have shown that, compared to whites, black and brown people experience unequal access to school and lower education level (*[*Andrade & Dachs, 2007*](https://doi.org/10.1590/S0100-15742007000200009)*), worse access to healthcare services (*[*Silva et al., 2020*](https://www.scielo.br/j/reben/a/nMTkjYhjBNwbqmQCDZNPKzM/)*;* [*Cobo et. al, 2021*](https://doi.org/10.1590/1413-81232021269.05732021)*), higher risk of disease (*[*Dantas-Silva et al., 2023*](https://www.ncbi.nlm.nih.gov/pmc/articles/PMC10281767/#:~:text=In%20Brazil%2C%20infection%20and%20death,those%20with%20low%20socioeconomic%20status.&text=Mortality%20from%20COVID%2D19%20was,other%20women%20in%20our%20country.)*), including mental disorders* [*(Filha et al, 2016)*](https://www.sciencedirect.com/science/article/pii/S0165032715306789?casa_token=-9LiNLPpFcUAAAAA:oTTEVKJn86ISD9uvp7XUhOi4rdJr6MFct26lR_n1AFP5_wGt8UaTXRkrDlMQZVTNZ58E5TWbP-o) *and higher mortality (*[*Oliveira et. al, 2020*](https://www.scielo.br/j/csp/a/QvQqmGfwsLTFzVqBfRbkNRs/?lang=pt)*;* [*Rebouças, et. al, 2022*](https://www.thelancet.com/journals/langlo/article/PIIS2214-109X(22)00333-3/fulltext)*). As pointed by Barber et al (2018), residential segregation also is an important structural determinant in racial health inequalities in urban areas in Brazil. According to the authors, blacks and browns are systematiclly and disproportionatelly exposed to highly segregated neighborhoods. Such living conditions were considered a fundamental cause for higher prevalence of cardio-metabolic diseases (diabetes and hypertension) among blacks and browns. From this perspective, understanding how these disparities reflect on the heat-related health risks today can contribute to the design of adaptation policies to reduce the exposure of vulnerable groups aligned with reducing social and economic inequalities."*

Lastly, the reviewer has commented that "based on DNA, there are no races, in the plural" and that "the use of phenotype is more appropriate than race." However, we would like to stress that the terms “race” and “ethnicity” are widely used in the recent IPCC Sixth Assessment Report - Impacts, Adaptation and Vulnerability ([Birkmann et al., 2022](https://www.ipcc.ch/report/ar6/wg2/chapter/chapter-8/)), in Chapter 8 (Poverty, Livelihoods and Sustainable Development):

1 - “(...) *intersections of age, gender, socioeconomic class,* ***ethnicity, and race*** *are recognized as important to the climate risks and differential impacts and losses experienced by vulnerable, marginal and poor in societies*”;

2- “(...) *anthropogenic climate change is hindering poverty alleviation and thereby constraining responses to climate change by threatening underlying gender inequalities exacerbated by climate impacts, such as access and control to productive inputs and reinforcing social-cultural norms that discriminate against gender, age groups, social classes, and* ***race***”;

3 - *“*(...) *social phenomena such as age, gender, class,* ***race and ethnicity*** *(...) shape social inequalities and experiences of the world and also intersect with climate hazards and vulnerability*”;

4 - *“*(...) *differentiation based on* ***ethnicity and race*** *leads to groups in society being less visible, with fewer rights, particularly for livelihoods that expose them to extremes. Unequal access to adaptation opportunities and benefits.”*

*5 - “Intersectionality recognizes that age, gender, class,* ***race, and ethnicity*** *are reinforcing social phenomena, shaping social inequalities and experiences of the world, and also intersect with climate hazards and vulnerability.”*

Moreover, to our best knowledge, no mention of the term “phenotype” appears in this chapter focused on the societal consequences of climate change and related hazards regarding adverse and irreversible effects for the most vulnerable, which is also our study's focus.

**References**

Andrade, C. Y. D., & Dachs, J. N. W. (2007). Access to education by age groups, according to income and race/skin colour in Brazil. Cadernos de Pesquisa, 37, 399-422. DOI: <https://doi.org/10.1590/S0100-15742007000200009>

Araújo, E. M. D., Costa, M. D. C. N., Hogan, V. K., Araújo, T. M. D., Dias, A. B., & Oliveira, L. O. A. (2009). The use of the variable of race/color within Public Health: possibilities and limits. Interface-Comunicação, Saúde, Educação, 13, 383-394. DOI: <https://doi.org/10.1590/S1414-32832009000400012>

Barber, S., Diez Roux, A. V., Cardoso, L., Santos, S., Toste, V., James, S., Barreto, S., Schmidt, M., Giatti, L., & Chor, D. (2018). At the intersection of place, race, and health in Brazil: Residential segregation and cardio-metabolic risk factors in the Brazilian Longitudinal Study of Adult Health (ELSA-Brasil). Social science & medicine (1982), 199, 67–76. <https://doi.org/10.1016/j.socscimed.2017.05.047>

Birkmann, J., E. Liwenga, R. Pandey, E. Boyd, R. Djalante, F. Gemenne, W. Leal Filho, P.F. Pinho, L. Stringer, and D. Wrathall, 2022: Poverty, Livelihoods and Sustainable Development. In: Climate Change 2022: Impacts, Adaptation and Vulnerability. Contribution of Working Group II to the Sixth Assessment Report of the Intergovernmental Panel on Climate Change [H.-O. Pörtner, D.C. Roberts, M. Tignor, E.S. Poloczanska, K. Mintenbeck, A. Alegría, M. Craig, S. Langsdorf, S. Löschke, V. Möller, A. Okem, B. Rama (eds.)]. Cambridge University Press, Cambridge, UK and New York, NY, USA, pp. 1171–1274, doi: <https://doi.org/10.1017/9781009325844.010>

Cobo, B., Cruz, C., & Dick, P. C. (2021). Gender and racial inequalities in the access to and the use of Brazilian health services. Ciência & Saúde Coletiva, 26, 4021-4032. <https://doi.org/10.1590/1413-81232021269.05732021>

Dantas-Silva, A., Santiago, S. M., & Surita, F. G. (2023). Racism as a Social Determinant of Health in Brazil in the COVID-19 Pandemic and Beyond. Revista brasileira de ginecologia e obstetricia : revista da Federacao Brasileira das Sociedades de Ginecologia e Obstetricia, 45(5), 221–224. DOI: <https://doi.org/10.1055/s-0043-1770135>

Filha, M. M. T., Ayers, S., da Gama, S. G. N., & do Carmo Leal, M. (2016). Factors associated with postpartum depressive symptomatology in Brazil: the Birth in Brazil National Research Study, 2011/2012. Journal of affective disorders, 194, 159-167. DOI: <https://doi.org/10.1016/j.jad.2016.01.020>

Hsu, A., Sheriff, G., Chakraborty, T. et al. Disproportionate exposure to urban heat island intensity across major US cities. Nat Commun 12, 2721 (2021). <https://doi.org/10.1038/s41467-021-22799-5>

Ma, Y., Zang, E., Opara, I. et al. Racial/ethnic disparities in PM2.5-attributable cardiovascular mortality burden in the United States. Nat Hum Behav (2023). <https://doi.org/10.1038/s41562-023-01694-7>

Muniz, J. O. (2010). On the use of the variable race-color in quantitative studies. Revista de Sociologia e Política, 18(36), 277-291. DOI: <https://doi.org/10.1590/S0104-44782010000200017>

Oliveira, R. G. D., Cunha, A. P. D., Gadelha, A. G. D. S., Carpio, C. G., Oliveira, R. B. D., & Corrêa, R. M. (2020). Racial inequalities and death on the horizon: COVID-19 and structural racism. Cadernos de Saúde Pública, 36. DOI: <https://doi.org/10.1590/0102-311X00150120>

Rebouças, P., Goes, E., Pescarini, J., Ramos, D., Ichihara, M. Y., Sena, S., ... & Paixão, E. S. (2022). Ethnoracial inequalities and child mortality in Brazil: a nationwide longitudinal study of 19 million newborn babies. The Lancet Global Health, 10(10), e1453-e1462. DOI: <https://doi.org/10.1016/S2214-109X(22)00333-3>

Silva, N. N. D., Favacho, V. B. C., Boska, G. D. A., Andrade, E. D. C., Merces, N. P. D., & Oliveira, M. A. F. D. (2020). Access of the black population to health services: integrative review. Revista Brasileira de Enfermagem, 73. <https://doi.org/10.1590/0034-7167-2018-0834>

Travassos, C., & Williams, D. R. (2004). The concept and measurement of race and their relationship to public health: a review focused on Brazil and the United States. Cadernos de saúde pública, 20, 660-678. <https://doi.org/10.1590/S0102-311X2004000300003>

**[R1.2]** In the eq. (1) show that Ti is corresponded to day 1, and i+1 to day 2 etc.

**[A1.2]** To clarify, we added the following sentence in the new version of the manuscript:

*"*Where *i* corresponds to the first day under HW.*"*

**[R1.3]** pg 15 'small time interval'? How small? The same for 'significant rise' at pg 16.

**[A1.3]** We now specify these values in the revised version of the manuscript:

*"Most of these events occur in short time intervals after the previous recorded HW* ***(<30 days).****"*

*"Also, Skansi et al. (2013) found a significant rise in extreme temperatures over Amazon, Northeast Brazil, and Southeast SA,* ***with increasing trends of 1.20, 3.05, and 0.44*** *in the number of warm days per decade, respectively, evidencing the warming across the whole SA since the mid-20th century onwards."*

**[R1.4]** Pgf from pg 18 is too long. Do a new one after the word 'overall'.

**[A1.4]** The paragraph was modified in the revised version of the manuscript accordingly.

**[R1.5]** pg 21 HW have occurred in winter ... Add winter/dry season. There is no 'winter' there.

**[A1.5]** Thanks for the suggestion. We modified the paragraph as suggested.

**Reviewer #2:**

**[R2 General Comment]:** We thank the authors for their changes to the paper. We still have a few outstanding issues that have not been addressed from our prior comments:

[A2-General Comment] We thank the reviewer for acknowledging the comprehensive changes we have included in the manuscript's revised version and for providing further comments. We have made an effort to address the remaining outstanding issues and improve the manuscript as follows:

**[prior R2-3]** – Thank you for addressing some of our comments. Please provide clarity regarding whether a complete case analysis was done, and the potential impact for the cities with high missingness of temperature data (e.g., Florianapolis).

**[A2.3]** In addition to the analyses presented in the last round of review, demonstrating that the missing data are uniformly distributed across both hot and cold months, we also assessed the temporal distribution over the years, and the data is spread throughout the entire historical series, ensuring there is no bias (e.g., a single year, whether exceptionally hot or cold, concentrating a significant portion of the missing data). Furthermore, for most of the MRs included in this study, the missingness of temperature data is below 10%, varying from 11% to 17% only in three of them (Salvador, Florianópolis, and Fortaleza), it's worth noting that the WMO Guide to Climatological Practices recommends a threshold of 20% for missing data, below which they are considered acceptable (WMO, 2018).

We improved the following sentence in the section 2.2 (Data) in the revised manuscript:

“*For temperature data, the percentage of missing values along the time series was below 10%, varying from 11% to 17% only in three of them (Salvador, Florianópolis, and Fortaleza), it is worth noting that the WMO recommends a threshold of 20% for missing data, below which they are considered acceptable (WMO, 2018). No significant differences were observed in missingness between cold and warm months, and missing data are spread throughout the entire historical series.*”

**References**

WMO. World Meteorological Organization. Guide to Climatological Practices. 2018 ed., World Meteorological Organization, 2018. ISBN: 978-92-63-10100-6

**[prior R2-4]** –Thank you for addressing some of our comments. Please clarify what is meant by non-natural confounding variables. Additionally, it is still unclear how missingness of demographics was ‘carefully addressed in the O/E ratio’? Clarify what was done with deaths missing race and education data – if they were excluded, please address potential biases (e.g., selection bias). Lastly, please describe how ill-defined deaths vary during heatwaves.

**[A2.4]** Thank you for raising these three issues that require further clarification. Below, we address each one individually:

a) We are sorry for using an unclear term in the previous answer to the reviewer's comment [R2-4]. Non-natural confounding variables mean deaths caused by accidents, suicide, and homicides. While the total number of deaths associated with these non-natural causes is usually relatively small, their inclusion influences the pure heat-related mortality analysis, as discussed in the previous round of answers. Therefore, only deaths associated with natural causes were considered, similar to the studies cited in the previous answer.

b) Regarding the missingness of race and education data, we agree with the reviewer's suggestion and have therefore modified the sentence in the new version of the manuscript to clarify, as follows:

*"To avoid any bias related to missingness, for both race and education data, we normalize the daily number of deaths in different categories (black/brown/white or low/high educational level), dividing by the number of records filled with respectively race/education information, and then multiplying by the total number of deaths."*

c) Regarding ill-defined deaths and unknown causes of mortality, as discussed in the previous round of revision, it is part of the ICD XVIII (Symptoms, signs, and abnormal clinical and laboratory findings, not elsewhere classified) and ranged from 1.3% to 9.3% for all MRs, except for Manaus, where it reached 16.6% of data. The increase in the number of deaths in ICD XVIII during HWs was analyzed in the first version of the manuscript and presented in the old version of Figure 6 (reproduced below). Most MRs had no significant variations in the number of deaths "not elsewhere classified" during heatwaves. In the highly populated MRs of São Paulo, Rio de Janeiro, and Belo Horizonte, there was a slight increase (O/E < 1.4), while in Porto Alegre, the increase was between 40 and 80% in the number of deaths. In all cases, it represented a small fraction of excess deaths (~5%). It should be stressed again that the discussion about ill-defined deaths (ICD XVIII) was removed from the original manuscript after the first round of revisions, following the Reviewer’s #1 firm disagreement on their inclusion (R1 General Comment).

**Old Figure 6** - The leading causes of death during excessive mortality HWs across the largest MRs in Brazil. The colors represent the range of O/E values, including blues (1 <O/E ≤1.4), greens (1.4< O/E ≤1.8), and reds (O/E> 1.8). The percentage of HW-related ED attributed to each cause of death is represented by the top color bars. Blank/not shown values are not statistically distinguishable (C.I. 95%).

**[prior R2-6]** – Thank you for addressing our comments. Please address which software was used for all analyses, not just the slope analysis

**[A2.6]** We added a sentence in the new version of the manuscript clarifying which software was used for data processing, quantitative analysis, and data visualization, as follows:

"Therefore, the O/E ratio and the corresponding confidence interval (C.I. 95%, as proposed by Hoshiko et al., 2010) were calculated, and, for events in which it was significantly higher than unity, the excess mortality was estimated. ***Data processing and quantitative analysis were performed using the Pandas open-source data analysis and manipulation tool, built on top of the Python programming language. Data visualization was performed using seaborn and matplotlib Python data visualization libraries.***"

**[prior R2-7]** – Thank you for addressing our comments. Please consider adding the purpose of the event-based surveillance analysis (rumor search) to the objective of the study.

**[A2.7]** We thank the reviewer for the suggestion. We have added a sentence in the Introduction section of the new version of the manuscript, with the purpose of the rumor search analysis, as follows:

*"Additionally, we performed an event-based surveillance analysis to investigate the early detection and monitoring of mortality outbreaks in Brazil during extreme temperature events. Such tool can be particularly relevant for public health strategies, similar to what has been developed in several countries (WHO, 2023)”*

Considering that event-based surveillance analysis pointed to the fact that heat is still a neglected disaster in Brazil, we also add the following sentence in the Conclusions:

*“Although the excess mortality estimated in this study (48,075 ED) was 20 times greater than the number of deaths associated with landslides nationwide (*[*Macedo and Sandre, 2022*](https://ipt.br/2023/03/29/mortes-por-deslizamentos-no-brasil-1988-a-2022/)*) in the same period, extreme heat is still a neglected disaster in Brazil, as pointed out by the results of our event-based surveillance analysis. A scenario further worsened by the growing population aging experienced by the Brazilian population in the last decade, according to the IBGE 2022 Census (people aged 60 or over raised from 11.3% to 14.7% of the population between 2012 and 2023).”*

Moreover, we accommodated the reviewer's suggestions, reinforcing in the abstract that our event-based surveillance analysis did not detect the HW-mortality nexus, which points out extreme heat events as a neglected disaster in Brazil.

**References**

WHO. World Health Organization. EIOS Newsletter - July 2023. Geneva: World Health Organization; 2023

**[prior R2-11]** –Thank you for including Table S1, but please interpret the uncertainty around the coefficients in the results (e.g., ICD XII has very wide confidence intervals). There are very wide confidence intervals in some of the ICD chapters.

**[A2.11]** We added the following paragraph in the discussion of the newly revised manuscript, interpreting the uncertainties around the coefficients:

"*Although uncertainties for most causes of death associated with the O/E values were smaller than 20%, certain ICD categories presented larger confidence intervals. Notably, ICD XII (Diseases of the skin and subcutaneous tissue) had uncertainties exceeding 90% of the O/E value in the MRs of Goiânia, Porto Alegre, Recife, Curitiba, and Brasília, besides O/E values indistinguishable from unity in the MRs of Manaus and Cuiabá. Similarly, ICD V (Mental and behavioral disorders) exhibited uncertainties above 40% of the O/E value in most MRs. For ICD V, excess deaths during HWs were not statistically significant in the MRs of Cuiabá, Fortaleza, Goiânia, and Manaus. The low total mortality counts for these specific diseases certainly influence large uncertainties for ICD V and XII, indicating the need for long-term studies focused on these specific causes in Brazil. Despite substantial uncertainties, excess mortality for these particular ICD chapters was statistically significant in some MRs, especially those with larger populations, and, thus, greater statistical significance. Regarding individual MRs, uncertainties in the O/E values were below 20% for most ICDs, except for the MR of Cuiabá, where uncertainties exceeded 25%, yielding non-significant results for ICDs XII and V, but also for ICD XIV (Genitourinary system diseases). The small population and the lower number of HWs compared to the other MRs can partially explain large uncertainties for the MR of Cuiabá.*"

**Reviewer #3:**

No Response
