# Supplementary material for: Twenty-first-century demographic and social inequalities of heat-related deaths in Brazilian urban areas
Source: PLoS One. 2024 Jan 24;19(1):e0295766. doi: 10.1371/journal.pone.0295766 (PMC10807764; doi:10.1371/journal.pone.0295766)
Supplement: Attachment — Submitted filename: PONE-D-23-10924 - Answer to reviewer comments.pdf [file pone.0295766.s004.pdf]

PONE-D-23-10924 - Answer to reviewer comments

Twenty-first-century demographic and social inequalities of heat-related deaths in Brazilian urban areas

To organize all individual comments and answers plus actions, we highlighted the comments and answers in the following way: Blue text shows the reviewer comments [R1], [R2, and [R3], and black text shows the authors' response [A1], [A2], and [A3].

Reviewer #1:

[R1 General Comment]: The manuscript is well written, tackling an important issue about heatwaves and mortality in the Brazilian major cities. However, it can be improved, following some suggestions given below:

First of all, why all diseases? Many of the cited diseases are rather complex with indirect weather impact, difficult to establish any correlation. Infection diseases are one of them where vectors and bacteria/viruses are involved. I strongly suggest modifying it, keeping the main diseases supported by literature such as respiratory, cardiovascular and maybe neoplasm. The latter is also complex since, for example, cancers caused by UV and stronger radiation, during HW, can be the triggering but not the final death. Heat waves have a short duration to present these impacts on death tolls.

[A1-General Comment] We thank the overall positive evaluation of the manuscript by the reviewer and the important suggestions. We partially agree with the reviewer and have removed a large number of diseases from the analysis. However, we kept the analysis of skin/subcutaneous and mental/behavioral disorders, diseases of the genitourinary, digestive, endocrine, respiratory, and circulatory system, neoplasms, and endocrine diseases, that show some level of statistical association with HWs in Brazil (see new Fig. 6). The example given by the reviewer has more than one possible rationale. It is possible (in fact likely) that terminally ill cancer patients die more frequently during intense HWs, not because of the long-term effect of UV radiation, but simply because, like so many other terminally ill people, they are too fragile to withstand the extreme heat of these events. This statistical link between HWs and cancer patients has been highlighted in previous assessments (Hertel et al., 2009; Huang et al., 2018). Likewise, previous analyses have found substantial increases in mortality and hospital admissions related to genitourinary, and nervous system diseases (Shaposhnikov et al., 2014) and diseases of the digestive system (Manser et al., 2013) during heatwaves. Section 4.4 presents a discussion in line with previous studies that have reported similar results related to the diseases that we kept in the analysis.

References in A1 General Comment

Hertel, S., Le Tertre, A., Jöckel, KH. et al. Quantification of the heat wave effect on cause-specific mortality in Essen, Germany. Eur J Epidemiol 24, 407–414 (2009). https://doi.org/10.1007/s10654-009-9359-2

Huang, C., Cheng, J., Phung, D., Tawatsupa, B., Hu, W., & Xu, Z. (2018). Mortality burden attributable to heatwaves in Thailand: A systematic assessment incorporating evidence-based lag structure. Environment international, 121, 41-50. https://doi.org/10.1016/j.envint.2018.08.058

Shaposhnikov, D., Revich, B., Bellander, T., Bedada, G. B., Bottai, M., Kharkova, T., ... & Pershagen, G. (2014). Mortality related to air pollution with the Moscow heat wave and wildfire of 2010. Epidemiology (Cambridge, Mass.), 25(3), 359. https://doi.org/10.1097/EDE.0000000000000090

Manser CN, Paul M, Rogler G, Held L, Frei T. Heat waves, incidence of infectious gastroenteritis, and relapse rates of inflammatory bowel disease: a retrospective controlled observational study. Am J Gastroenterol. 2013 Sep;108(9):1480-5. doi: 10.1038/ajg.2013.186. Epub 2013 Aug 13. PMID: 23939628.

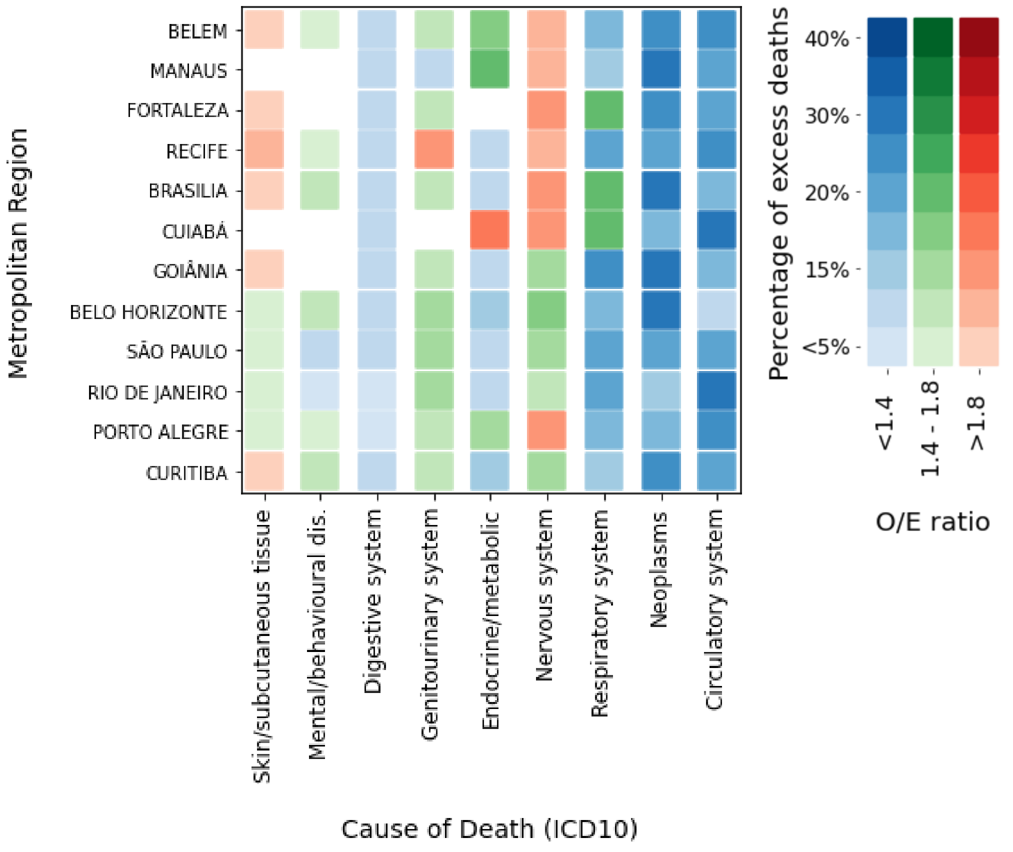

New Version of Figure 6 - The leading causes of death during excessive mortality HWs across the largest MRs in Brazil. The colors represented the range of O/E values, including blues (1 <O/E ≤1.4), greens (1.4< O/E ≤1.8), and reds (O/E> 1.8). The percentage of HW-related ED attributed to each cause of death is represented by the top color bars. Blank/not shown values are not statistically distinguishable (C.I. 95%).

[R1 Minor comments]

[R1-1] The last paragraph of the Introduction section, starting with 'Accordingly', should be separated in a new paragraph.

[A1-1] Thanks for the suggestion. In the revised manuscript we have separated the last paragraph of the Introduction section accordingly.

[R1-2] São Paulo City means the Metropolitan Area of Sao Paulo, not only the city itself. The same for Rio.

[A1-2] Thanks for the comment. Throughout the manuscript, we only use MRs of São Paulo and Rio de Janeiro, instead of São Paulo city or Rio de Janeiro city. The only exception can be found in the introduction, when we cite a multicountry study by Bell et al. (2008) we used "São Paulo City" because this was a city-level study.

[R1-3] Black/white dichotomy: Brazilian population is rather blended. According to Pena et al (2009), the 'racial' mixture is so high that cannot be used for medical proposals. Therefore, add a paragraph about it, since the results are interesting.

[A1-3] We thank the reviewer for the comment and reference, however, we emphasize that there are several studies on different fields in public health that consider the racial split in Brazil (e.g. Kabab et al., 2012; Guimarães et al., 2022; Santos Ferreira and Camargo, 2021). Most of them have shown that race is a strong predictor of variability in mortality (Chor and Lima, 2005), irrespective of socioeconomic status (Renna Junior, et al., 2021). We also add a paragraph about that accordingly.

References in A1-3

Kabad, J. F., Bastos, J. L., Santos, R. V. (2012). Race, color and ethnicity in epidemiologic studies carried out with Brazilian populations: systematic review on the PubMed database. Physis, 22(3), 895. https://doi.org/:10.1590/S0103-73312012000300004.

Chor, D., & Lima, C. R. D. A. (2005). Epidemiologic aspects of racial inequalities in health in Brazil. Cadernos de Saúde Pública, 21, 1586-1594. https://doi.org/10.1590/s0102-311x2005000500033.

Guimarães, M. N. et al (2022). Racial Inequities in Self-Rated Health Across Brazilian Cities: Does Residential Segregation Play a Role?, American Journal of Epidemiology, Volume 191, Issue 6, June 2022, Pages 1071–1080, https://doi.org/10.1093/aje/kwac001

Renna Junior, N. L., Lima, C. A., Laporte, C. A., Coleman, M. P., & e Silva, G. D. A. (2021). Ethnic, racial and socioeconomic disparities in breast cancer survival in two Brazilian capitals between 1996 and 2012. Cancer Epidemiology, 75, 102048. https://doi.org/10.1016/j.canep.2021.102048

Santos Ferreira, R. B., & de Camargo, C. L. (2021). Vulnerability of the Black Population in Brazil to the Evolution of the COVID-19 Pandemic. rev. cuid.(Bucaramanga. 2010), 1-12. https://doi.org/10.15649/cuidarte.1322

[R1-4] equation 1. explain Ti. Why did not use max temperatures? I also suggest modifying it to include that variable. Mean temperature smooths a lot the heat wave impacts.

[A1-4] We thank the reviewer for commenting on the EHF metric. Some authors have been working with different types of HW metrics in the last two decades. Initially, we would rely

mostly on indices based on Tmax temperatures (e.g. Garcia et al., 2005). However, after the outstanding 2003 HW in Western Europe, it became obvious that besides daytime Tmax we had to consider also the effect of nighttime Tmin impacts (Trigo et al., 2009). In this context, several works have been using HW indices that consider both Tmax and Tmin temperatures. Here, in accordance with several previous studies that have assessed the temperature-mortality relationship, we choose to use the EHF parameter, which is a previously defined indicator of heat-related human health impacts (Oliveira et al., 2022) that considers both the Tmax and Tmin to quantify the daily heat-stress conditions (Nairn and Fawcett, 2015). This parameter has been largely used and proved to be adequate to characterize the meteorological conditions when the aim is to analyze the temperature-mortality link Langlois et al., 2013; Nairn and Fawcett, 2013, 2014; Scalley et al., 2015; Hatvani-Kovacs et al., 2016b; Jegasothy et al., 2017a; Nairn et al., 2018).. According to the authors:

"Daily maximum temperatures are only part of the story when considering impacts on human health, agriculture, infrastructure, the demand on utilities (water, electricity, etc.), and other environmental hazards such as fire. Previous research has highlighted the importance of incorporating minimum temperature through the utilization of daily mean temperature, a line of thought we follow here. The extent to which heat is dissipated overnight following a very hot day dictates the accumulating thermal load impacting vulnerable people and systems. The accumulation of this heat which is not being dissipated overnight results in “excess heat”.

Furthermore, by using the same metric as in previous studies, our results can be more easily interpreted and compared with previous findings and results obtained for several regions including Brazil (Geirinhas et al., 2020).

Accordingly, we improve the methods sections including the following sentence:

"Through the utilization of DMT, instead of only daily maximum temperature, the EHF index incorporates minimum temperature. According to Nairn and Fawcett (2015), thermal accumulation depends on the extent to which heat is dissipated during the night. Accumulation of undissipated heat results in overheating. After a very hot day, this excess determines the load that impacts vulnerable people."

References

Geirinhas, J.L., Russo, A., Libonati, R. et al. Heat-related mortality at the beginning of the twenty-first century in Rio de Janeiro, Brazil. Int J Biometeorol 64, 1319–1332 (2020). https://doi.org/10.1007/s00484-020-01908-x

Oliveira, A., Lopes, A., & Soares, A. (2022). Excess heat factor climatology, trends, and exposure across European functional urban areas. Weather and Climate Extremes, 36, 100455.

Langlois, N., Herbst, J., Mason, K., Nairn, J., & Byard, R. W. (2013). Using the Excess Heat Factor (EHF) to predict the risk of heat related deaths. Journal of forensic and legal medicine, 20(5), 408-411.

Nairn, J., & Fawcett, R. (2011). Defining heatwaves: heatwave defined as a heat-impact event servicing all. Europe, 220, 224.

Nairn, J. R., & Fawcett, R. J. (2015). The excess heat factor: a metric for heatwave intensity and its use in classifying heatwave severity. International journal of environmental research and public health, 12(1), 227-253.

Scalley, B. D., Spicer, T., Jian, L., Xiao, J., Nairn, J., Robertson, A., & Weeramanthri, T. (2015). Responding to heatwave intensity: excess heat factor is a superior predictor of health service utilisation and a trigger for heatwave plans. Australian and New Zealand journal of public health, 39(6), 582-587.

Hatvani-Kovacs, G., Belusko, M., Pockett, J., & Boland, J. (2016). Can the excess heat factor indicate heatwave-related morbidity? A case study in Adelaide, South Australia. EcoHealth, 13, 100-110.

Jegasothy, E., McGuire, R., Nairn, J., Fawcett, R., & Scalley, B. (2017). Extreme climatic conditions and health service utilisation across rural and metropolitan New South Wales. International journal of biometeorology, 61, 1359-1370.

Nairn, J., Ostendorf, B., & Bi, P. (2018). Performance of excess heat factor severity as a global heatwave health impact index. International journal of environmental research and public health, 15(11), 2494.

[R1-5] Mij is the observed number of deaths, right? Add it.

[A1-5] That's right. We add that information to the original phrase:
"... Mij is the observed ***number of deaths*** *over all HW days. Expected mortality is calculated considering the average* ***number of deaths*** *over periods …"*

[R1-6] Explain what 'rumor' exactly means and give more details how the data can be used in section 2.5.

[A1-6] We improved section 2.5 in the revised version with the definition of the meaning of "rumors", according to the World Health Organization (WHO, 2014), and a short description of the Epidemic Intelligence from Open Source (EIOS):

"A rumor consists rumor of any information circulating, usually from unofficial sources, about reports of disease outbreaks, adverse events, impacts of health products or services, or other information about a negative effect on health (WHO, 2018; Zhang et al., 2020; Grein et al., 2000; Velasco et al., 2014; Palagyi et al., 2019; Morgan et al., 2022; Williams et al., 2021)"

Providing a more precise definition, an important reviewer's suggestion, allows a better interpretation of the event-based surveillance analysis results (see R1-9 and R2-7).

References in A1-6

Morgan, O.W., Abdelmalik, P., Perez-Gutierrez, E., Fall, I.S., Kato, M., Hamblion, E., Matsui, T., Nabeth, P., Pebody, R., Pukkila, J., Stephan, M., Ihekweazu, C., 2022. How better pandemic and epidemic intelligence will prepare the world for future threats. Nat. Med. 28, 1526–1528. https://doi.org/10.1038/s41591-022-01900-5

Williams, G.S., Impouma, B., Mboussou, F., Lee, T.M.-H., Ogundiran, O., Okot, C., Metcalf, T., Stephen, M., Fekadu, S.T., Wolfe, C.M., Farham, B., Hofer, C., Wicht, B., Tores, C.C., Flahault, A., Keiser, O., 2021. Implementing epidemic intelligence in the WHO African region for early detection and response to acute public health events.

World Health Organization. Regional Office for the Western Pacific. (2008). A guide to establishing event-based surveillance. WHO Regional Office for the Western Pacific. https://apps.who.int/iris/handle/10665/207737

ZHANG L, et al. How the Health Rumor Misleads People's Perception in a Public Health Emergency: Lessons from a Purchase Craze during the COVID-19 Outbreak in China. Int J Environ Res Public Health. 2020 Oct 2;17(19):7213. doi: 10.3390/ijerph17197213. PMID: 33023103; PMCID: PMC7579116.

GREIN TW et al. Rumors of disease in the global village: outbreak verification. Emerg Infect Dis. 2000 Mar-Apr;6(2):97-102. doi: 10.3201/eid0602.000201. PMID: 10756142; PMCID: PMC2640857.

VELASCO, Edward et al. Social media and internet-based data in global systems for public health surveillance: a systematic review. The Milbank Quarterly, v. 92, n. 1, p. 7-33, 2014. Available in: https://doi.org/10.1111/1468-0009.12038

PALAGYI, Anna et al. Health system preparedness for emerging infectious diseases: a synthesis of the literature. Global Public Health, v. 14, n. 12, p. 1847-1868, 2019.

[R1-7] ED popped out without a proper introduction. It means expected deaths, right?

[A1-7] Thanks for catching this typo. It means excess deaths. In the revised version of the manuscript we now introduce the term correctly the first time it appears in the text: *"In Brazil, around 1800* ***excess deaths (ED)*** *were related …."*

[R1-8] During the sections of Results there are many 'highly than' and similar without any values to support such statements. Or at the end of 3.5 section without percentiles. Please provide.

[A1-8] Thanks for the comment. Accordingly, we added those values in the results sections (3.1, 3.2, 3.3, 3.4, and 3.5) of the revised manuscript.

[R1-9] The last sentence of section 3.6: 'none rumors were found', that means no media shows that? Please clarify.

[A1-9] Thanks for pointing this out. We've improved the sentence in the revised manuscript, and additionally, as mentioned before (see the response to R1-6) we now provide a more complete definition of what a rumor is: *"Despite the increasing number of rumors detected with EIOS, none of them matched any of the high-mortality HWs presented in Table 4, even though they were the events in which the largest increase in mortality (highest O/E ratio) was observed in each MR."*

Discussion.

[R1-10] Please provide a range of the HW trends among the cities. The results show different behavior.

[A1-10] We provided the HW trends among the cities in Table 2. Accordingly, we now add the range of HW trends in section 3.1, as follows: *"Statistically significant (C.I. 95%) increasing trends for HW frequency were observed in most MRs (except for MRs of Rio de Janeiro and Porto Alegre) (Table 2)****, ranging from 0.06 HW year^{-1} (Florianópolis) to 0.36 HW year^{-1} (Belém),*** *with high values also in Manaus (0.23 HW year^{-1}) and Goiânia (0.20 HW year^{-1})."*

[R1-11] 'Significant rise of ...' gives numbers and an explanation why, since the climate is already hot.

[A1-11] In this paragraph we contextualized our results, comparing them with previous studies conducted in South America, particularly in Brazil. We reported a significant rise in warm days observed by Skansi et al. (2013). To clarify, we added in the revised manuscript the numbers as suggested by the reviewer: *"Also, Skansi et al. (2013) found a significant rise in extreme temperatures over Amazon, Northeast Brazil, and Southeast SA****, with increasing trends of 1.20, 3.05 and 0.44 in the number of warm days per decade, respectively, evidencing the warming across the whole SA since the mid-20th century onwards.****"*

[R1-12] 2006-2100 is spelled wrongly: 2006-2010.

[A1-12] The text was corrected accordingly.

[R1-13] Another point that should be stressed. All data is based on deaths per 100,000 or so inhabitants, right? It is a percentile, not the absolute number because the population in each category is different.

[A1-13] That's correct. Since the population in each category is different, the excess mortality estimates were presented in percentage and/or observed-to-expected ratio values. We added this information in section 3.3 of the revised manuscript.

[R1-14] Despite the fact that the authors cited humidity as a gap in the manuscript, I also suggest including it (see Fernanda Diniz thesis, 2022), because high humidity can overload the elderly metabolic capacities.

[A1-14] We thank the referee for the comment and reference. As stated before (when answering R1-4), we relied critically on the use of the EHF metric that is based on temperature variables alone. As discussed in R1-4, the EHF index has been shown to be more sensitive to the relationships between health and heat than current heat wave indices. Moreover, although we acknowledge the relevance of humidity (as in the heat index (IC) used in Fernanda Diniz's thesis 2022), we opted to keep the index as simple as possible and compatible with previous studies (such as Geirinhas et al., 2020, and others cited when answering R1-4).

[R1-15] Figure 6 displays another source.

[A1-15] We modified Fig. 6 (see the answer to R1 General Comment).

Reviewer #2:

[R2 General Comment]: This article focuses on exploring demographic and socioeconomic differentials in susceptibility to the effects of heat waves in 14 Large Brazilian cities. The authors use an index to quantify the duration and intensity of heat waves, and then calculate a measure of excess mortality during these heat waves. They find heterogeneity in the impacts of heat wave by city, and stronger effects among the elderly, women, racial/ethnic minorities and individuals of low educational level. I have a few concerns I have outlined below:

[A2-General Comment] We thank the overall positive evaluation of the manuscript by the reviewer.

[R2 Major comments]:

[R2-1] Introduction: the authors frame the need for their study around a lack of studies exploring heterogeneities in the effects of heat on health, especially in Latin America (and in Brazil in particular). First, I'd like to point out is that the Kephart 2022 study they cite does explore regional heterogeneity within Brazil, as it shows results of rate ratios for each Brazilian city, showing how tropical areas are generally less sensitive to heat (due to lower temperature variation). This other paper by the same group (https://www.sciencedirect.com/science/article/pii/S0277953622008322) looks at social and demographic characteristics of cities in relationship with these heterogeneities. Second, I think the authors should cite more comprehensive evidence regarding the lack of evidence in Latin America. A good paper to cite may be this one by Berrang-Ford (https://www.nature.com/articles/s41558-021-01170-y) where the authors find a lack of research in the region.

[A2-1] Thanks a lot for the recommendation. We totally agree with the reviewer. However, we would like to mention that the study by Bakhtsiyarava et al. (2023) was published after the first submission of this manuscript. Nevertheless, we agree that it is paramount to include this reference in the revised version of the manuscript. Accordingly, we improved the third paragraph of the introduction taking into account the comments and the important additional references recommended by the reviewer, as follows:

"Despite the increasing number of global studies investigating heat-related mortality risk using Brazilian cities (Vicedo-Cabrera et al., 2021), there is still a huge knowledge gap on the role played by regional, demographic, and socioeconomic aspects ***(Berrang-Ford et al., 2021).*** *This represents a particular challenge in Brazil, considering the continental size of the*

country, the variety of different climates to be considered, and a predominantly urban population that is rapidly aging. ***As an example, Kephart et al. (2022) found lower changes in mortality under extreme heat in the tropical areas in northeast Brazil with relatively low temperature variability, compared to temperate areas of southern Brazil. Another recent study by Bakhtsiyarava et al. (2023) investigated the association between socioeconomic and demographic characteristics and temperature-related human mortality in Latin American cities. According to the authors, income inequality increases mortality from cold events for all ages, in addition to segregation and poverty, which are associated with higher excess mortality due to cold among the elderly. However, for heat-related mortality, the study reveals an opposite behavior, with higher levels of poverty and income inequality associated with lower heat-related mortality, which highlights the need for further investigation, given the increasing importance of urban adaptation to climate change, as discussed by the authors.***"

[R2-2] Setting: how are MRs defined? I assume there's an IBGE definition, but this is not mentioned.

[A2-2] We thank you for pointing out this missing information in the manuscript. The definition used was in accordance with the IBGE, so we have updated the text: *"In the present study, we considered the 14 most populous Brazilian MRs,* ***as defined by the IBGE****"*

[R2-3] Data: the authors indicate some percent of missing values, but it isn't clear whether this corresponds to temperature or mortality. In any case: what's the pattern of this missingness? Is it more common in warmer months? It is unclear how the missing data was handled. Did authors conduct a complete case analysis, excluding all missing data?

[A2-3] The percentage of missing data indicated in the original manuscript corresponds only to temperature data. Regarding the missing pattern of the data, we thank the reviewer for pointing out this important aspect to be evaluated. To do this, we calculated the percentage of missing temperature data in each MR, for all years in the range 1961 to 2020 and grouped them into warm (Jan, Feb, Mar, Oct, Nov and Dec) and cold months (Apr, May, Jun, Jul, Aug and Sep). As shown in the figure below (figure 2-3), although the percentage of missing data varies between RMs, no significant differences in missingness are observed between cold and warm months.

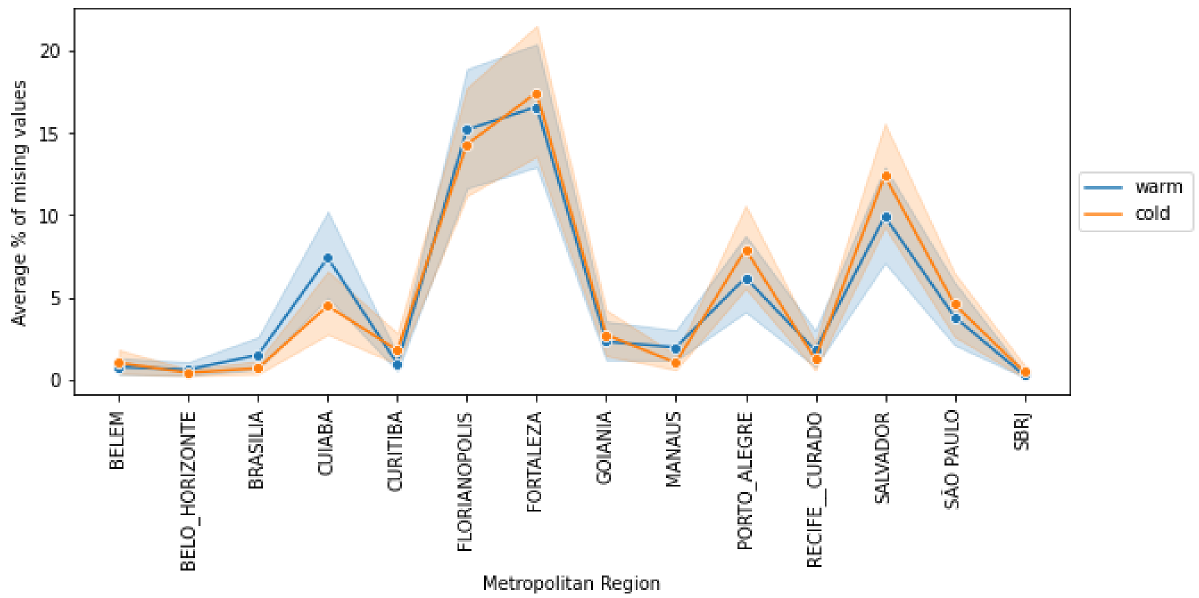

Figure 2-3 - Percentage of missing temperature data in each MR, for all years in the range 1961 to 2020 and grouped into warm (Jan, Feb, Mar, Oct, Nov, and Dec) and cold months (Apr, May, Jun, Jul, Aug, and Sep). The solid line represents the average over all years, the shaded area represents the standard deviation.

Accordingly, we improve the sentence in the revised version of the manuscript:

*"**For temperature data,** the percentage of missing values along the time series was below 5% for most of the MRs, except for the MRs of Cuiabá (6%), Porto Alegre (7%), Salvador (11%), Florianópolis (15%) and Fortaleza (17%), not affecting the long-term characterization of HWs. **Furthermore, no significant differences in missingness are observed between cold and warm months.**"*

Regarding missingness on mortality data, we discuss that when answering the following question (R2-4).

[R2-4] Mortality data specifically: what's the justification for excluding injuries? There's some research showing increases in homicides with heat, for example (https://pubmed.ncbi.nlm.nih.gov/28687898/). If the missing data %s in the data section correspond to temperature, what's the missingness in mortality data? I know education and race tend to have higher %s of missing data. Speaking of race: what happens with yellow and indigenous groups? (as I think they are called in Brazil) They seem to be excluded from this article (I understand their sample size may be small, but I'd mention explicitly if these are excluded deaths). Another potential source of missing data are ill-defined deaths or garbage

codes, which the authors don't seem to be addressing (and which is important especially if the number of these types of deaths changes in heat waves).

[A2-4] We recognize that the increase in suicides, homicides, and other violence indicators with heat has been relatively well reported in recent years. However, in order to exclude any non-natural confounding variables that might influence the pure heat-related mortality analysis, only deaths associated with natural causes were considered, similar to the majority of previous studies for Brazil (Geirinhas et al., 2020; Bell et al., 2008), Argentina (Chesini et al., 2019), as well as in review studies at the global scale (Ng et al., 2016; Åström et al., 2011; Bell et al., 2008, among others). Despite that, as discussed in the answer to the R1 General Comment, we kept a relatively large number of diseases in our analysis often not explored in the literature (against the suggestion of reviewer 1 that asked explicitly to evaluate only the main three).

Regarding missing information on death records, overall the percentage of missing data is low for age (ranging from 0.1% in Florianópolis to 2.0% in Fortaleza) as well as for sex (ranging from 0.0% in São Paulo to 0.2% in Salvador), however, race and education present a higher percentage of missing data. This is due to the fact that these two categories of information have an additional "ignored" field with a high number of records, compared to gender and age. For race, missingness was below 10% for most of the MRs, ranging from 1.73% in Belém to 8.82% in Curitiba, with the exception of Fortaleza, reaching 13.8%. Regarding yellow and indigenous groups, according to the IBGE 2010 Population Census, they represent less than 2% of the population in Brazil, and for this reason, they were not included in the analysis. For education, the percentage of missing data was larger, ranging between 10% and 30% for most of the MRs. Higher values were observed for Recife (35.2%), Porto Alegre (42.1%), and Goiânia (52.1%), which is reflected in large uncertainties in the results for these MRs. The issue of missingness was carefully addressed in calculating the O/E ratio. For each category (sex, race, age, education), the number of deaths was normalized by the total number of deaths filled in (see equation 4 of the original manuscript), making the observed and expected values comparable for the daily number of deaths. We also point out that the MRs of Salvador and Florianópolis were not included in the stratified analyses.

Regarding ill-defined deaths and unknown causes of mortality, it is part of the ICD XVIII (Symptoms, signs, and abnormal clinical and laboratory findings, not elsewhere classified), ranging from 1.3% to 9.3% for all MRs, with the exception of Manaus, were the reaching 16.60% of data. The high proportional mortality from unspecified and ill-defined causes of death in the State of Amazonas (where the MR of Manaus is located) has already

been reported by a previous study (Balieiro et al., 2019), showing to be associated with space-time dimensions, demographic factors and socioeconomic status and medical assistance at the time of death. However, the authors also showed a significant reduction in the fraction of ill-defined causes in the state between 2006 and 2012.

Accordingly, we have added the following sentences in the revised version of the manuscript:

"Regarding death records, the percentage of missing data was low for age (ranging from 0.1% in Florianópolis to 2.0% in Fortaleza) and sex information (ranging from 0.0% in São Paulo to 0.2% in Salvador). However, race and education presented a higher percentage of missing data, mainly due to the higher number of records filled as "ignored" in this categories, compared to gender and age. For race, missingness was below 10% for most of the MRs (ranging from 1.73% to 8.82%) with the exception of Fortaleza (13.8%). For education, the percentage of missing data was larger (between 10% and 30% for most of the MRs), being higher for Recife (35.2%), Porto Alegre (42.1%), and Goiânia (52.1%), which reflects the large uncertainties in the results for these MRs. Regarding ill-defined deaths and unknown causes of mortality (ICD XVIII), it ranged from 1.3% to 9.3% for all MRs, with the exception of Manaus, were the reaching 16.60% of data. The high proportional mortality from unspecified and ill-defined causes of death in the State of Amazonas (where the MR of Manaus is located) has already been reported by a previous study (Balieiro et al., 2019), showing to be associated with space-time dimensions, demographic factors and socioeconomic status and medical assistance at the time of death. However, the authors also showed a significant reduction in the fraction of ill-defined causes in the state between 2006 and 2012."

"Missingness was carefully addressed in calculating the O/E ratio. For each group (sex, race, age, education), the number of deaths was normalized by the total number of deaths filled accordingly, in order to calculate expected number of deaths and the excess mortality."

References in A2-4

Ng, C. F. S., Boeckmann, M., Ueda, K., Zeeb, H., Nitta, H., Watanabe, C., & Honda, Y. (2016). Heat-related mortality: effect modification and adaptation in Japan from 1972 to 2010. Global environmental change, 39, 234-243.

Geirinhas, J.L., Russo, A., Libonati, R. et al. Heat-related mortality at the beginning of the twenty-first century in Rio de Janeiro, Brazil. Int J Biometeorol 64, 1319–1332 (2020). https://doi.org/10.1007/s00484-020-01908-x

Chesini, F., Abrutzky, R., & Titto, E. D. (2019). Mortality from heat waves in the city of Buenos Aires, Argentina (2005-2015). Cadernos de Saúde Pública, 35.

Åström, D. O., Bertil, F., & Joacim, R. (2011). Heat wave impact on morbidity and mortality in the elderly population: a review of recent studies. Maturitas, 69(2), 99-105.

Bell, M. L., O'neill, M. S., Ranjit, N., Borja-Aburto, V. H., Cifuentes, L. A., & Gouveia, N. C. (2008). Vulnerability to heat-related mortality in Latin America: a case-crossover study in Sao Paulo, Brazil, Santiago, Chile and Mexico City, Mexico. International journal of epidemiology, 37(4), 796-804.

Balieiro, P. C. D. S., Silva, L. C. F. D., Sampaio, V. D. S., Monte, E. X. D., Pereira, E. M. D. S., Queiroz, L. A. F. D., ... & Costa, A. J. L. (2019). Fatores associados à mortalidade por causas inespecíficas e mal definidas no estado do Amazonas, Brasil, de 2006 a 2012. Ciência & Saúde Coletiva, 25, 339-352. https://doi.org/10.1590/1413-81232020251.27182017

[R2-5] Exposure: The authors use what seems to be like a widely used metric of heat waves, the excess heat factor or EHF. This is calculated based on two indices: (1) a significance index, which is the difference between a 3-day running mean of daily temperature vs the 95th percentile of historical (between 1981-2010—why specifically those years?) temperature over the whole year; and (2) an acclimatization index, which is the difference between this 3-day running mean and the 30-day mean temperature. Both indices are then multiplied, taking the raw significance index and modifying it by the acclimation index. This EHF, if positive, indicates a heat wave, and is then compared to all positive EHFs (I assume historically? For the same city? Unclear from the text), and if it falls below the 85th percentile, the heat wave is considered low intensity, if it falls above 3x the 85th percentile it'd be extreme, and otherwise it'd be severe. If this explanation is incorrect, there may need to be some clarification done in the text. What is the reasoning behind differentiating between severe and extreme heat waves if they were interpreted as a single category?. Furthermore, is there any temporal restriction conducted here? For example only considering summers for the cities where this makes sense (non equatorial)? How are cold temperatures considered? Does this index already take that into account, since the EHF would be negative in those cases?

[A2-5] We thank the reviewer for this large comment related to exposure and the metric of heat waves. Regarding the EHF computation, we used the period between 1981-2010 as reference climatological period, because that is the climatological standard normal period

recommended by the WMO Guidelines on the computation of Climate Normals (WHO, 2017).

The positive EHF indicates a day under heat wave conditions and we compare it to all positive EHFs historically and for the same city. The reviewer's explanation is correct. We differentiate between severe and extreme heat waves to be in agreement with the method described by Nairn and Fawcett (2015) and widely used in heat-related mortality studies since we didn't propose any new methods. However, we interpreted it as a single category given the low number of extreme heat waves. Moreover, we didn't find significant differences between these two categories in terms of impacts.

We also add a sentence in the methods section:

"*Despite we used the method described by Nairn and Fawcett (2015) to classify HWs, in some parts of this paper, severe and extreme events will be interpreted as a single category, given the low number of extreme HWs and similar results regarding impacts on mortality.*"

As proposed by Nairn and Fawcett (2015), there is no seasonal temporal restriction on the EHF computation, however, as a consequence of the definition of EHF, most heat waves are detected during the summer in non-equatorial regions. All temperatures are considered, including cold temperatures. In this case, when temperatures are low, the EHF becomes negative (no heat wave), and therefore it is not taken into account for the calculation of EHF85 (the metric for heat wave intensity).

We add a sentence in the methods section:

"*Days with negative EHF (i.e. no HWs) are not taked into account for the calculation of EHF85*".

Reference in A2-5

WMO. (2017). WMO guidelines on the calculation of climate normals. WMO Technical Report.

Nairn, J.R.; Fawcett, R.J.B. The Excess Heat Factor: A Metric for Heatwave Intensity and Its Use in Classifying Heatwave Severity. Int. J. Environ. Res. Public Health 2015, 12, 227-253. https://doi.org/10.3390/ijerph120100227

[R2-6] Excess mortality estimation: in calculating the O/E ratio, the authors compare actual mortality during each heat wave (Mij) to a historical average in previous and subsequent

years. But is this average conducted using the same days of the year? And what do the authors mean by “Reference periods under HW condition, either in an earlier or later year, were not included in the calculation of expected mortality.”? Does this mean that if a day fell under a HW, they wouldn’t include it here? Last, there’s no mention to what software was used for all statistical analysis.

[A2-6] Regarding the historical average, yes, it was conducted using the same Julian days of the year. Regarding days excluded from the reference period your assessment is correct, i.e. if those days in the reference period (i.e. same Julian days in previous and subsequent years) fall under an HW event, they are excluded from the computation of expected mortality in order to capture only the expected mortality under non-heatwave conditions. This methodology is often used, particularly when the total number of years available to compute the reference mortality is relatively small (Trigo et al., 2009).

To clarify this aspect we improve the paragraph as follows: *"... over periods of the same duration* ***using the same Julian days of the year*** *in previous (M_{i1}, M_{i2},..., $M_{i,j-1}$) and subsequent ($M_{i,j+1}$, …, $M_{i,k-1}$, $M_{i,k}$) years, ranging from 1 (in this case representing the year 2000) to k (2018). Reference periods under HW condition* ***(i.e. days that fell under a HW)****, either in an earlier or later year, were not included in the calculation of expected mortality."*

Reference in A2-6

Trigo, R. M., Ramos, A. M., Nogueira, P. J., Santos, F. D., Garcia-Herrera, R., Gouveia, C., & Santo, F. E. (2009). Evaluating the impact of extreme temperature-based indices in the 2003 heatwave excessive mortality in Portugal. environmental science & policy, 12(7), 844-854.

[R2-7] Rumor search: what is the purpose of this section in the methods section? (2.5). How is a ‘rumor defined?I don’t see this mentioned in the objectives or justified in the introduction, and it doesn’t seem to be connected to the rest of the analysis (at least insofar one can find this information in the methods section). This seems to be used in section 3.6 to identify whether the most impactful heat waves were actually picked up by media. However, while an interesting finding, this is seems to be out of the scope of this paper, and would need way more detail, justification, statistical testing, etc, to be part of these analysis in my opinion.

[A2-7] We thank the reviewer #2 for the positive evaluation of the findings obtained through the rumor search. We already provided more details about the meaning of “rumors” and how to conduct a rumor search in methods and results in agreement with the comments of the reviewer #1 (please see R1-6 and R1-9). Additionally, as suggested by the reviewer #2, we improve this aspect of the manuscript, as follows:

Introduction: "*In this sense, in addition to death due to HW, other adverse effects on health can be minimized by adopting measures for the early identification of risk situations. For this, the World Health Organization (WHO) recommends event-based surveillance, a strategy that uses the capture and analysis of unofficial information, published by users of social media, as a source of alert (WHO, 2008; 2014).*"

Methods: "*An event-based surveillance analysis using rumor search was performed using Epidemic Intelligence from Open Source (EIOS), which is a tool implemented by WHO to identify HW rumors on social media (WHO, 2008; 2014), n order to identify if the HWs registered in Brazil were mentioned in some rumor on media. A rumor is any information circulating, usually from unofficial sources, about reports of disease outbreaks, adverse events, impacts of health products or services, or other information about a negative effect on health (WHO, 2008; Zhang et al., 2020; Grein et al., 2000; Velasco et al., 2014; Palagyi et al., 2019). EIOS, through internet-based media monitoring, gathers epidemic intelligence information from different sources, including traditional online media and specific social media sources, government and official websites, news aggregators, blogs and expert groups and collaboration, among others. The system automatically collects, filters and processes news from relevant online sources, categorizing them based on key terms, languages, regions, time periods, etc. In the risk management strategy of public health emergencies and in the surveillance process, electronic tools have been increasingly used as monitoring and early warning mechanisms (WHO, 2014; Morgan et al., 2022; Willians et al., 2021). The period covered by the search was 2000-2018, using the terms "heat waves" + "Brazil" + "health", all news categories, all languages, and placing only Brazil in the filter for the country cited.*"

[R2-8] Descriptive results: Figure 2 is great, see some comments in the minor comments section. I think table 2 would benefit from adding the confidence intervals to the slope, instead of underlining the nonsignificant ones (if I understand the footnote correctly). Moreover, this slope analysis does not seem to be described in the methods section, so it is unclear to me how the trend was calculated. Also, the headings on Table 2 are a little unclear. Why were the frequency of HW events assessed through '20s (does this include through 2022?) while the duration of HWs only through '10s. Why was the decade of the 1990's completely skipped over? The authors may want to be more specific and just indicate the ranges (e.g. 2000-2020 instead of "2000's/2010's")

[A2-8] As suggested by the reviewer, in the revised version of the manuscript we added the confidence interval of the slope in Table 2. We also provided a brief description of the slope analysis in the methods section: ***"From the HWs calculated using the EHF index, we***

determined the total annual number of events for each MR and performed a trend analysis using the Ordinary least squares Linear Regression method with the scikit-learn tool, a free software machine learning library for the Python programming language". Regarding the headings, we now specify the ranges in Table 2, instead of decades. The 1990s were skipped over in order to compare extremes in the time series (first 2 decades versus last two decades), thus visualizing the remarkable increase in the number and duration of the events. We emphasize that the quantitative analysis, however, is expressed through the slope, which includes all years of the complete time series.

[R2-9] Main results: table 3 and figure 3 outline the main results. I suggest the authors sort this table and figure in the same way as Table 2 and Figure 2, to not confuse the reader. The figure shows the O/E ratios (although it's missing the confidence intervals, which would be tricky to show here, but which could be shown in a similar figure as Figure 6 here (https://read.dukeupress.edu/demography/article/55/4/1363/167907/Bayesian-Estimation-of-Age-Specific-Mortality-and). In table 3, it'd be great to see confidence intervals around the excess deaths to know how much variability there's around these estimates. Last, for figure 3, since the y-axis is a ratio, it should be plotted in the log scale (given that ratios can go from 0 to infinity, and therefore the distance from 0 to 1 should be the one as from 1 to infinity [or, to use a more realistic example: distance of 0.5 and 1 should be the same as 1 to 2]).

[A2-9] The MRs in Table 3 are sorted by the total excess deaths (i.e. from 2000 to 2018). In agreement with the suggestion of the reviewer, we sorted Table 3 and Figure 3 in the same way in the revised manuscript. However, in the case of Figure 2 and Table 2, we opted to keep sorting the MRs by latitude, in order to evidence the clear latitudinal regional differences in the increasing trends of HWs in Brazil (see R2-16). In Table 3, we added the confidence intervals around the excess deaths, as suggested by the reviewer. We also modify Figure 3, plotting the O/E ratio in the log scale, which is more appropriate, as suggested by the reviewer. As mentioned by the reviewer, it is very tricky to show the confidence intervals of each individual O/E value, since there are several points in the graph, however, this information is indirectly presented by the color of the dots (red indicates that the O/E ratio is statistically higher than 1), as well as in the new version of Table 3, by adding the confidence interval of excess deaths as suggested by the reviewer.

[R2-10**]** Stratified results: Figure 4 and 5 show the results stratified by age, sex, education and race, showing that elderly people, women, and people of low education and black/brown people have higher ED due to heat. A few comments: (1) the first part of Figure 4 is hard to interpret without normalizing by the number of people in each age group; (2) for both the

second part of figure 4 and all of figure 5, I wonder if the authors could compute a ratio between women/men, black/brown and white, high and low education, to know what the excess risk is (And confidence intervals around those ratios); (3) as with Figure 3, both figure4b and figure 5 should be plotted in the log scale (see explanation above).

[A2-10] Thanks for raising these issues in order to improve the overall quality and readability of figs. 4 and 5. Concerning comment 1, we chose to keep Figure 4 as it is, because we believe that the percentage of heat-associated deaths stratified by age group (as a percentage in total excess deaths) provides direct and easier-to-assimilate information for identifying the most vulnerable groups and designing public health strategies. However, we thank the reviewer for the comment and agree that these results need to be interpreted considering the proportion that each category represents, not only in the population, but also in the total number of deaths observed in the period analised. Taking it into account, we add a figure with the percentage of total deaths observed in each age group between 2001 and 2018 (see Fig S2 in SM). The number of deaths in older and eldest groups (>65) varies from 47.65% in the MR of Manaus to 59.90% in Rio de Janeiro. These numbers are much lower than the percentage of heat-related ED that we observed, reinforcing what was pointed out by the reviewer, i.e. when normalized, the proportion of deaths in the oldest groups is even higher. Accordingly, we add a sentence in the discussion on stratified results, as follows:

"The large fraction of excess heat-related deaths among the elderly people over 65 years (75-94% of excess deaths), contrasts with the distribution of total mortality from natural causes (Fig. S2). When considering all natural deaths in the period from 2001 to 2018, the older and eldest groups represent less than 60% of the observed mortality, ranging from 47.65% in the MR of Manaus to 59.90% in the MR of Rio de Janeiro."

Regarding comment 2, it's a great suggestion. We calculated the excess risk range for MRs where the difference between women/men, black/brown and white, and high and low education was significant, and added a paragraph about it on discussion, as follows:

"Disparities in the risk of heat-related death associated with race and education were also observed across the MRs. For males over 65 years, excess mortality for low education sub-group was 10% (3%-18%) higher than for those with high-educational level in the MR of São Paulo, 22% (12-33%) in Rio de Janeiro, 42% (17-71%) in Porto Alegre, 53% (30-80%) in Belém and 96% (56-147%) in Recife. Likewise, the excess risk for 65 years old and older females low-educational level subgroup was 8% (0-17%) in São Paulo, 24 (13-36%) in Rio de Janeiro, 36% (13-63%) in Belém, 62% (27-107%) in Recife, and 62% (16-128%) in Manaus, compared to low-education. In relation to race, the difference in excess mortality between black/brown people and white people was 10% (2-19%) in Rio de Janeiro, 15%

(0-31%) in Belo Horizonte, 17% (9-25%) in São Paulo, 27% (0-60%) in Brasília, 33% (0-78%) in Manaus 40% (14-72%) in Recife, 44% (25-67%) in Belém, 65% (31-106%) in Goiânia, and 92% (17-213%) in Curitiba, for males with >65 years. For females (65 years and older), the results for the heat-related mortality increase among blacks and browns was 8% (2-16%) higher than for whites in the MR of Rio de Janeiro, 8% (1-15%) in São Paulo, 34% (11-62%) in Recife, 41% (22-63%) in Belém, 60% (25-104%) in Brasília, 82% (41-135%) in Goiânia, 203% (1-409%) in Cuiabá."

Finally, we plotted Figures 3, 4b, and 5 in the log scale, as suggested by the reviewer.

[R2-11**]** Results by cause of death: this section would greatly benefit from considering confidence intervals. I see a few causes of death that are very rare (e.g. skin and subcutaneous tissue) with very high O/E ratios, potentially because of very low mortality rates to begin with. By addressing significance, or at least acknowledging variability around the O/E, the authors can focus on the more meaningful results. Additionally, authors should consider defining what 'not elsewhere classified' means. Is this related to missing data or ill-defined codes? What % are not classified?

[A2-11] We agree with the reviewer that considering confidence intervals in results by cause of death can greatly benefit this section, however since Figure 6 shows both the O/E ratio and the percentage of excess death attributed to each ICD for each MR, it's very tricky to add the information about the confidence interval in the Figure 6. Thus, in the revised version we opted to add some extra information (number of deaths expected and observed, excess deaths, O/E ratio with the respective confidence interval) in the SM. Regarding 'not elsewhere classified', deaths as discussed in the answer to the R1 General Comment, we have removed a large number of less heat-sensitive diseases from this analysis, including this specific ICD. Nonetheless, we also add a discussion about diseases not elsewhere classified (ill-defined deaths and unknown causes of mortality) (see answer R2-4).

[R2-12**]** The results by specific heat wave (table 4) are interesting, but have not been detailed in the methods section. Again, confidence intervals would help here, along with a normalization of deaths by population (the high numbers of Sao Paulo and Rio de Janeiro may be just because of the size of these cities

[A2-12] The specific heat waves presented in Table 4 are the ones with the large increase in mortality (i.e. the higher O/E ratio). We add a sentence in the methods section accordingly (*"based on the values of O/E ratio, we identified the HWs with the large mortality increase for each MR, and classified these events according to the excess deaths, duration and*

intensity, and the interval after previous HW for further analysis."). We also improved Table 4, now including the confidence intervals for the O/E ratio and number of excess deaths.

We agree with the reviewer that the number of deaths in São Paulo and Rio de Janeiro tends to be larger due to the high population, however, the HWs presented in Table 4 were selected based on the O/E ratio, i.e. the greatest increase in excess mortality, which is already normalized since is a relative increase. We opt to present the absolute daily number of deaths per day to allow an discussion more aligned with the analysis of rumors. The results showed that, despite the high number of excess deaths (absolute numbers, not normalized), these events were generally not detected, highlighting the need for health surveillance strategies during heat waves. Furthermore, the population of all MRs is provided in the manuscript and all results are discussed always considering the different populations of these regions.

[R2-13] The authors have written a good discussion, contextualizing results with prior studies and providing what seems to be adequate interpretation. The only part I think I'm missing is a more methodological discussion comparing the results to articles that look at temperature overall (instead of heat waves, for example using distributed lag non-linear models), those that look at cold vs hot temperatures, etc. It may be helpful to understand the rationale for the absence of a lag period in the analysis. Additionally, the discussion may benefit from addressing why most of the excess mortality events occurred during low-intensity heat waves rather than during severe/extreme heat waves.

[A2-13] Thanks for the comment. Regarding the absence of a lag period, we agree that it can be regarded as a limitation of our paper, as it can underestimate the mortality effects of HWs. Despite being a simple method, the EHF calculation considers a three-day-averaged daily mean temperature and somehow adds a longer period into the HW. However, we recognize this as a limitation. This aspect was also pointed out by reviewer #3 and we have added in the revised version a discussion on that issue (see R3-3). Regarding the events with large increase in mortality, we improved the discussion, as follows:

"This result can be partially interpreted in line with the EHF definition considering that if a HW is preceded by another HW within an interval of less than 30 days, this implies a lower value of the EHF acclimatization index, which results in a HW classified as low intensity according to the definition. However, in this case, the acclimatization effect does not happen smoothly. On the contrary, a temperature spike of a few days is responsible for a rise in the 30-day temperature average (used in calculating the EHF acclimatization index). In addition, recent studies have shown that the impacts of consecutive hazards can significantly weaken the health situation, as there is a greater chance of increasing the strain on both the health

service and the health of the population due to repeated extreme heat. The prevalence of these consecutive disasters is increasing due to climate change, also increasing socioeconomic exposure and vulnerability (de Ruiter et al., 2020)."

"The predominance of low-intensity HWs among the events with the largest increase in mortality also indicates that the use of EHF for classifying HWs in terms of intensity may not be adequate for the regional reality in Brazil, which certainly motivates future research on the construction of new indices that better represent the impact of heat on the health of the population in Brazilian areas."

References

de Ruiter, M. C., Couasnon, A., van den Homberg, M. J., Daniell, J. E., Gill, J. C., & Ward, P. J. (2020). Why we can no longer ignore consecutive disasters. Earth's future, 8(3), e2019EF001425.

[R2 Minor Comments]

[R2-14] Abstract: the abstract points to both older folks/women and low educational level/black/brown people having a higher susceptibility to heat, but the sentence where this is shown makes it seems like these correspond to two different analyses. Are they part of the same result?

[A2-14] Thanks for the comment. We reformulated the sentence in the Abstract accordingly: *"Higher heat-related excess mortality was observed for low-educational level, black and brown people, older and females."*

[R2-15] Figure 1: while I appreciate the very complete descriptive figure, the Koppen climate figure is a bit confusing, as it includes all of South America, with unclear borders of where Brasil is. My intuition is that the thicker lines are Brazilian states, but since they aren't showed anywhere else in this figure it's hard to know where Brazil ends in this figure. As it is, it gives the impression of wider climate heterogeneity (as it includes the high altitude regions of Peru, Bolivia and Chile, and the desert regions of Chile and Argentina. Furthermore, it isn't clear to me what the pink bars are (they look to be bars indicating population, but we lack axes, and the information of population is already there).

[A2-15] We modify the figure accordingly

[R2-16] Figure 2: I commend the authors for sorting the cities meaningfully (by latitude). However, it'd be great to include a label that makes this obvious to the reader. For example, since Belem is around the equator, you could include a line next to Belem and label it

Equator, with an arrow pointing up (North) and one pointing down (South). In any way, this is an excellent figure! It shows increasing number of heat waves and intensity, especially in the South.

[A2-16] We thank the reviewer for the positive comments about Figure 2. We already add this information in the legend of the figure, but we also modify the figure accordingly to make this obvious to the reader.

Reviewer #3:

[R3 General Comment] The analysis describes long-term trends and excess deaths from heat waves in 14 large metropolitan areas in Brazil over a period of decades. While other studies have examined the impacts of hot temperatures on mortality, this analysis helpfully provides findings on the impacts of consecutive hot days (i.e. heat waves), which plausibly have distinct, cumulative effects on physiological stress and death. The analysis of variability in excess deaths by sex, age, race, and educational attainment is important and useful for policymakers. The paper could be improved by addressing the following points of confusion and by acknowledging some major limitations of the analysis which are not sufficiently described.

[A3-General Comment] We thank the overall positive evaluation of the manuscript by the reviewer.

[R3 Major comments]

[R3-1] The analysis excludes deaths from external causes (e.g. accidents and homicide), but the authors' describe the analyzed deaths as "all-cause" throughout the manuscript. It is more common to call non-accidental deaths "non-injury deaths" or "natural deaths" or similar, instead of calling them all-cause deaths. All-cause deaths typically mean truly all causes, without excluding deaths from external causes.

[A3-1] Thank you for your comment. The reviewer is correct and therefore we fixed this expression in the revised manuscript. The term "all-cause" was used specifically in the legend of Table 3, and clarified accordingly: "*HW-related excess* ***deaths (excluding external causes)*** *over the 2000–2018 period and regarding the…*"

[R3-2] The authors do not sufficiently discuss the interaction between age and gender in which subpopulations have increased risk. Older adults have greater risk, and women have greater risk, however, we know that older adults are disproportionately women because a greater proportion of men have already died. The manuscript briefly mentions that elderly women may be more likely to live alone and therefore have higher risk, but more attention needs to be given to the large sex differences in the age distribution of the population, particularly at older ages. If the authors could add analyses of sex differences stratified by age that would be helpful. If not, they should soften the description of the findings on sex disparities and add discussion of the limitation of not knowing whether this is due to survival bias or due to biological or environmental differences in exposure or response to heat. The policy implications are large, so this is an important nuance to present correctly.

[A3-2] We agree that this is an important aspect that needs to be addressed correctly. We take this opportunity to stress that initially we disaggregated the combined age and sex groups, i.e. older men, older women, adult men, adult women, etc. The results were statistically significant for MRs with large populations, such as São Paulo and Rio de Janeiro. However this multiple division into several subgroups implied relatively small samples of data for most MRs (Salvador and Florianópolis, for example, were removed in a large part of the segregated analyses). We compared segregated analyzes (women in different age groups, men in different age groups) with non-segregated analyzes (women total, men total) and the results in the cities where it was possible to perform (São Paulo, Rio de Janeiro, Belo Horizonte, for example) were identical within the confidence interval. For this reason, to keep the RMs and analyzes as consistent as possible, we chose not to include sex differences stratified by age.

We add the following paragraph in the discussion:

"Another issue in this sense is the absence of analyzes of sex differences stratified by age. Although we observed higher mortality rates among women, it is hard to quantify whether this is due to the women/men survival bias or due to biological or environmental differences in exposure or response to heat. Since policy implications are very large, we recommend further investigation into this particular aspect."

And the following paragraph in the methods:

"We also conducted analyzes of sex differences in heat-related mortality stratified by age. Despite the results were statistically significant for the MRs with large populations, the division of death records into several subgroups reduced the dataset for most MRs, making the analysis unfeasible. Moreover, the results comparing analysis segregated by sex and

age with non-segregated analyzes for MRs with a large population, the results were comparable within the confidence interval. Therefore, we chose not to include this analysis stratified by sex and age."

[R3-3] The modeling approach (as described) does not include any lags in the effects of heat on subsequent mortality. This is a major limitation of the paper, as other analyses from around the world have consistently demonstrated mortality effects 1-4 days after heat exposure. Given this major limitation, it is likely that the mortality effects described in this manuscript are underestimates of the true mortality effects, since deaths on subsequent days are not considered. This deserves a notable discussion in the manuscript.

[A3-3] We agree that our assessments do not include any lag effect that could represent a major limitation as this can underestimate the mortality effects of HWs. Nonetheless, as discussed in the previous answer, the EHF calculation considers a three-day-averaged daily mean temperature, and thus including intrinsically a longer period into the HW. However, we recognize this as a limitation of the work and have added a sentence about it:

"As discussed before, a limitation of this paper is that it does not include any lags in the effects of heat on mortality, particularly since other studies have demonstrated mortality effects during 1-3 days after exposure (Huang et al., 2014). As such, our approach can underestimate the mortality effects and, therefore, suggest that the number of heat-related excess deaths can be higher in most of the MRs. We point out however that, since the EHF calculation considers a three-day-averaged daily mean temperature. and that somehow adds intrinsically a more extended period to the HW."

Reference

Huang J, Wang J, Yu W. The lag effects and vulnerabilities of temperature effects on cardiovascular disease mortality in a subtropical climate zone in China. Int J Environ Res Public Health. 2014 Apr 11;11(4):3982-94. doi: 10.3390/ijerph110403982. PMID: 24733034; PMCID: PMC4025023.

[R3-4] The authors hypothesize in Section 4.5. that some low intensity HWs make have had high impact because they occurred a few weeks after other HW events. However, wouldn't this conflict with the assumptions in the HW assimilation metric, which assumes that heat within the past 30 days has an assimilating effect on the population?

[A3-4] We thank the reviewer for the interesting question. We agree that, according to the EHF metric, heat within the past 30 days has an assimilating effect on the population. However, the definition itself reinforces and helps to understand our hypothesis. If a HW is preceded by another HW within an interval of less than 30 days, this implies a lower value of the EHF acclimatization index, which results in a HW classified as low intensity by definition. However, in this case, the acclimatization effect does not happen smoothly. On the contrary, a temperature spike of a few days is responsible for a rise in the 30-day temperature average (used in calculating the EHF acclimatization index). In addition, as discussed in the R2-13 the impacts of consecutive hazards have been shown to be larger than the sum of different single events, increasing exposure and vulnerability (see R2-13). We add this discussion in the revised version of the manuscript, in session 4.5 (see R2-13).

[R3-5] I did not find Figure 3 to be useful. Consider moving to the supplement or removing. Since this focus is on cumulative risk, not isolating specific events, the jittered dots of individual HW events are not very informative to the reader. There is also no real need to have the dual-axis plot. Consider breaking into two panels.

[A3-5] We appreciate the reviewer's comment on figure 3. As pointed out by reviewer 2 (see R2-9), figure 3 outlines the main results regarding the increment of mortality and excess deaths during HW events in the MRs included in this study. Regarding the dual-axis plot, we chose to plot this information together (O/E ratio and Excess Deaths) just to make the figure more compact and visually clean. In this way, the two pieces of information can share the same x-axis, with the name of the MRs, making it easier to read the figure.

[R3-6] Education variables: 1) the Methods describe two categories, low and high, however these do not seem to include the entire range of educational attainment. How were these categories chosen and what happened to individuals who fell between the low and high education categories? It is not clear. 2) Was this data included in the individual death records or is this area level data based on residence?

[A3-6] We thank the reviewer for the comment. The number of education years is a variable included in the individual death records from Brazilian *DATASUS* database. We have inserted this information in the new version of the manuscript. Regarding education subgroups, as described in the Methods section, we used low class for those with less than four years of education, and high for 12 or more years of education. Although this division results in a reduction in the number of death records (since it removes individuals who fell between the low and high education categories), after a sensitivity test we observed that this makes the results more robust, as it allows us to compare extreme categories with each other. For clarity, we have modified/added to the text the following sentences:

"… besides the cause of death (according to the 10th revision of the International Statistical Classification of Diseases and Related Health Problems, ICD-10), educational level and race. All this information is provided by DATASUS in the individual death records. Educational level was divided into low (less than four years of education) and high (12 or more years of education). Although this division results in a reduction in the number of death records (since it removes individuals who fell between the low and high education categories), after a sensitivity test we observed that this makes the results more robust, as it allows us to compare extreme categories with each other."

[R3-7] Temperature data: Please comment on general patterns of where the meteorological stations were located within the metropolitan regions. In the urban core? Airports? Outside of the city? This is important for readers to assess how well the single monitor in each metro area may reflect true population exposures.

[A3-7] We added a sentence highlighting that all meteorological stations are located inside the urban core of the metropolitan regions included in this study: *"All meteorological stations are located inside the urban core of each MR."*

[R3-8] Race: please justify the choice of combining black and brown into one category. The authors describe how three groups are often used in research in Brazil, but the final variable appears to be a binary.

[A3-8] Thanks for the suggestion. We improved the manuscript, justifying the choice of combining black and brown into one category, as follows:

Regarding racial classification, the ternary system composed of blacks, browns ('pardos'), and whites has been used in previous studies on racial inequalities in health in Brazil (Barber et al., 2018), unlike the binary Black-White dichotomy most used in countries like the USA. ***However, according to article IV of Federal Law nº 12.288 of 2010, which creates the Statute of Racial Equality, the "Negros" are: "the group of people who declare themselves black or brown, according to the color or race used by the Brazilian Institute of Geography and Statistics Foundation (IBGE), or that adopt analogous self-definition". In Brazil, particularly in urban areas, the "Negros" are the group of the population that most live in conditions of greater deprivation of services, such as sanitation, higher rates of violence, unemployment, inadequate housing, etc., being disproportionately affected by social and environmental injustices (Baptista and Santos, 2022). Accordingly, we combine the black and brown into one category."***

[R3-9] Supplement: I did not receive a supplement, though it is referenced in the manuscript.

[A3-9] We are sorry for this inconvenience. We will attach the revised version of the SM to the review files.
